# Prevention Actions of Burnout Syndrome in Nurses: An Integrating Literature Review

**DOI:** 10.2174/1745017901915010064

**Published:** 2019-03-29

**Authors:** Sidney Medeiros de Oliveira, Luiz Vinicius de Alcantara Sousa, Maria do Socorro Vieira Gadelha, Vânia Barbosa do Nascimento

**Affiliations:** Faculty of Medicine of Santo André, São Paulo SP, Brasil

**Keywords:** Burnout, Nurses, Health promotion, Prevention and control, Psychooncology, Stress

## Abstract

**Aims:**

To identify the strategies for the prevention of burnout syndrome in nurses; and discuss the results for future interventions that can decrease burnout in these professionals.

**Design:**

An integrative review of the literature.

**Data Sources:**

PubMed, Lilacs, Medline, Scielo, and Science Direct, from April 2018 to July 2018.

**Methods:**

The sources were in all 553 references were found. The following guiding question was: Which interventions for the prevention of burnout in nurses have been applied and have obtained high effectiveness?

**Results:**

Based on the inclusion and exclusion factors, 30 studies were selected for analysis. The studies were categorized in individual, group and organizational, being the studies with actions in groups those of greater prevalence.

**Conclusion:**

The actions used to cope with burnout were, for the most part, effective, with some demonstrating greater success than others. From the 30 reviewed studies, the results did not obtain satisfactory improvement in burnout in only three interventions: 1) Systematic nursing supervision; 2) Basic nursing care; and 3) Psycho-oncological training program.

## 1 INTRODUCTION

Competitiveness at work has been identified as the main cause of stress in the work environment. Statistics show that one in five people may suffer from some form of mental health problem at work. Problems such as these have a direct impact on worker productivity, causing absenteeism, fatigue, and a decrease in the quality of service delivery. Currently, burnout syndrome is a great psychosocial problem, and has been causing concern on the part of researchers and health institutions, owing to the seriousness of its consequences at the individual and organizational levels [1]. In the United States, the term “burnout” emerged in the 1970s when Herbert J. Freunderberger (1974) realized that many of the volunteers with whom he worked had a decreased mood, with a later demotivation, and then physical and mental exhaustion. It was during this period that hypotheses began to emerge on the increase in pressures and, consequently, labor stress, especially in health professionals, such as doctors, nurses, and nursing technicians [2]. In the 1980s, psychologists Cristina Maslach and Susan Jackson began to study the emotions of health professionals in the work environment and defined “burnout” as physical and emotional fatigue resulting in a decrease or loss of motivation for work that can evolve to total exhaustion and a sense of failure [3]. In 1982, they created the Maslach Burnout Inventory with the intention of evaluating three main components: depersonalization, reduction of personal fulfillment, and emotional exhaustion of the individual. Subsequently, research on burnout syndrome has gained traction, being investigated in several countries, such as Germany, Spain, Italy, Canada, France, Israel, Poland, Sweden, and England [3].

## 1.1 Background

Burnout syndrome is the continuous exposure to work-stress associated with poor working conditions, in which pleasure and work performance decrease [4, 5]. In view of this, it is considered a multi-causal pathology, as it is related to excessive exposure to prolonged stress; it has identified aspects related to several stressors in the work environment that imply the commitment of the worker’s health triggering the disease, evaluated according to three components: depersonalization, exhaustion emotional, and professional achievement [6-8].

According to the World Health Organization, mental health can be considered as a state of well-being in which the individual is able to use their own resources and skills to recover from the stress of everyday life, without compromising productivity [1]. Stress is a system of adaptation of the individual to any unforeseen situation and prepares them for a quick and effective action [9].

In the health area, nursing stands out as one of the most exhausting professions owing to different circumstances in professional practice causing physical and emotional exhaustion. The nurse is expected to perform patient care with patience and empathy, all in a highly stressful environment, with few resources and an excessive workload, thereby requiring nurses to find a balance between these factors that interfere in their working life [7].

Thus, nursing is one of the most stressful occupations. It is common to find burnout syndrome in health professionals, especially in the field of nursing [10]. Some professionals manage to deal with the symptoms, but those who do not adapt to the long-term working conditions, insufficient number of professionals, and poor communication tend to feel physically and emotionally worn out [11]. For nurses, burnout reduces the ability to provide care. Every day, nurses face the dilemma of being human, empathetic, and sensitive, in a work environment of many responsibilities [7]. Situations discovered by professionals in patients, such as costly recovery or non-recovery, as well as the lack of capacity to deal with dark situations, such as death, can create a feeling of impotence and professional dissatisfaction [5]. Therefore, burnout prevention in health professionals, including nurses, has an important significance in promoting the physical and mental health of these service providers [11]. Burnout affects personality, performance, and productivity at work. The emotional responses that the disease can cause in the long run lead to a mental strain that will hardly neutralize spontaneously [12, 13]. In this context, this study aimed to analyze the actions of prevention and control in the workplace to reduce burnout in nurses.

The world health organization states that, most companies worldwide are more concerned with investment in medical treatments, when the recommendation is that they should invest more in prevention and in improving the quality of the work environment, to protect the well-being and health of workers. The symptoms of burnout syndrome are characterized initially by the inability of the individual to cope with stressful work situations [1].

The considerable professional turnover that is verified in some sectors and institutions makes the results appear limited when it comes to interventions in this area [14]. Recognition of other mental pathologies that can influence the development of burnout is crucial [15]. Symptoms of distress, coping, work limitations, job satisfaction, use of substances to relieve stress, alcohol consumption, and understanding of depression and anxiety are some of the points to be considered [16].

## 2 THE REVIEW

### 2.1 Aims

This investigation aimed to identify the prevention strategies of burnout syndrome in nurses; and discuss the results for future interventions that can increase the promotion of the health of nurses and decrease burnout in this class of professionals.

### 2.2 Design

The research adopted the model for an integrative review of literature. It is a methodology that has been highlighted, in relation to other research methods, since the mid-1980s as a research method for gathering information from several studies on the same subject, to reduce research gaps. Over the years, other definitions and suggestions have been incorporated into this method, making it one of the most effective in the search for knowledge for clinical practice, especially in the area of nursing (Mendes, Silveira, & Galvão, 2008).

### 2.3 Search Methods

In view of the above, the following guiding question was formulated: Which interventions for the prevention of burnout in nurses have been applied and have obtained high effectiveness? To acquire the data, some previously selected eligibility criteria were observed: (1) studies that approached the subject matter of the research; (2) published in any language; (3) in the period from 1994 to 2018; (4) any methodology applied to measure burnout before and after interventions. The research used the acronym PICO referring to the following: P (Population), for nurses; I (Actions investigated), for prevention and control; C (Comparative), for other studies; and O (Outcomes), for burnout. The time period, between 1994 and 2018, was selected to cover greater scope for discussion on the prevention and reduction of burnout and health promotion in nurses. The research period is justified; although the definition of burnout syndrome emerged in the 1970s, studies on this theme intensified after 1994.

The PubMed, Lilacs, Medline, Science Direct, and Scielo databases were used, from April 2018 to July 2018, with the following MeSH descriptors: “Burnout”, “Nurses”, “Health Promotion”, “Prevention and control”.

Their related terms in the English language were included. To make the search more secure, the Boolean operator AND was adopted. The Boolean AND operator enabled greater delimitation of the subject, allowing for greater intersection between the articles obtained. It was used as follows: Burnout AND Nurses AND Health promotion, and so on.

For the selection of the studies, screening and eligibility steps were carried out. The articles were initially evaluated by the titles and summaries for the identification of the inclusion criteria. For this stage, two authors worked independently in the search and selection of the studies through an initial reading of titles and abstracts. In the case of disagreement, a third reviewer was used to verify the situation of eligibility. Subsequently, a full analysis of each selected article was performed.

The variables analyzed for data extraction from the studies were author and year, place of study, number of professionals, interventions in groups, follow-up time, outcomes, and limitations. The final inclusion of articles was carried out in a judicious way by the full reading of the articles. After reading the articles in their entirety, the authors divided the articles into subgroups for an analysis of the most effective procedures in health promotion and the consequent decrease of burnout in nurses.

### 2.4 Search Results

The database search yielded 553 references: 82 in PubMED, 145 in Lilacs, 247 in Science Direct, 29 in Scielo, and 50 in Medline. After analyzing them according to the eligibility criteria, 30 studies were selected, as shown in Fig. (**1**).

### 2.5 Quality Appraisal

For the selection of the studies, screening and eligibility steps were carried out to include the articles in this review. The articles were evaluated by the titles and summaries initially for the identification of the initial inclusion criteria. For this, the pairing was used in which two authors worked independently in the search and selection of the studies by the initial reading of titles and abstracts and in the discordance a third reviewer was used to verify the situation of eligibility of the study. Subsequently, a full analysis of each selected article was performed.

### 2.6 Data Extraction

The following data were extracted for the research: Author and year, country of study, sample, method used, and search results.

### 2.7 Data Synthesis

In all, 553 articles were identified. Their abstracts were read and, using inclusion and exclusion criteria, a total of 30 articles were selected for complete reading. Of the 30 articles selected, 2 were from Sweden, 1 from the Netherlands, 2 from Canada, 10 from the United States (one in association with Israel), 4 from Brazil, 3 from Turkey, 1 from England, 2 from Israel (one in association with the United States), 2 from Japan,1 from South Africa, 1 from Australia, 1 from South Korea, and 1 from Iran.

## 3 RESULTS

The database search yielded 553 references: 82 in PubMED, 145 in Lilacs, 247 in Science Direct, 29 in Scielo, and 50 in Medline. After analyzing them according to the eligibility criteria, 30 studies were selected.

**Fig. (2):**
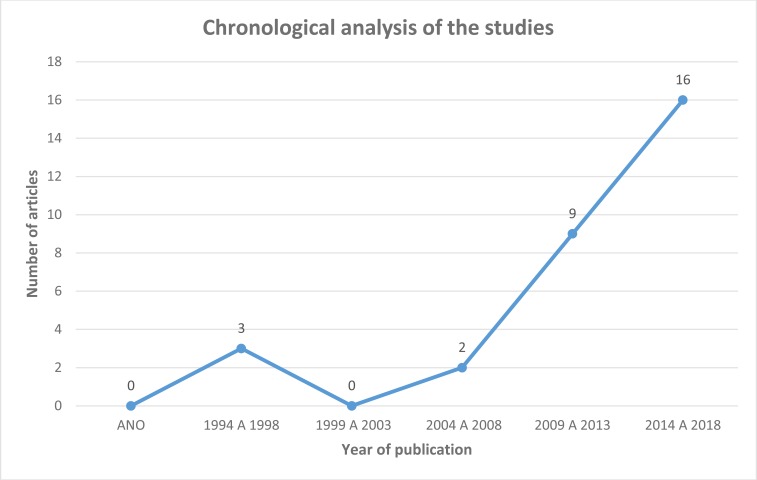
Chronological analysis of selected studies for research.

The chronological analysis of the selected studies (Fig. **2**) showed that, for the year of publication, the articles that were part of the sample behaved as follows: from 1994 to 1998, three articles; from 1999 to 2003, no article; from 2004 to 2008, 2 articles; from 2009 to 2013, nine articles; from 2014 to 2018, 16 articles. Thus, in the last 10 years, there is greater concern with the research on burnout in nurses.

Table **1** shows the various ways of coping with burnout according to the studies. Several methods were used to prevent or combat the stress that nurses face in their work environment. The types of intervention identified in the surveyed articles were as follows: yoga, cognitive coping strategies, compassion fatigue program, systematic clinical supervision, meditation, web-based stress management program, mental and the Psychological Empowerment Program.

The selected articles were categorized as focused on the individual, group, and organization, according to adaptations of the descriptions of Salanova and Llorens (2008) [17]. In the individual category, the interventions aimed at improving and qualifying personal internal resources through active behavior on the part of the individual. The interventions were grounded on empowering nurses through knowledge and the development of new skills. In the group category, the interventions aimed to improve communication, interpersonal relationships, and teamwork, to result in better patient care and developing resilience. In the organizational category, the objective was prevention with a focus on education, health promotion, and improvement and strengthening of the resources of social collectives. The idea was to break the isolation, improve the processes of socialization, and empower social support. The interventions in this category of studies also turned to the context-based self-reflection of the individual, emphasizing actions of cooperation.

Table **2** shows a higher prevalence of group or group interventions for prevention and control of burnout compared with individual or organizational approaches, with seven studies with individual interventions, 16 studies with group interventions, and seven studies with organizational interventions. Notably, all of the studies reported a concern to offer solutions to combat burnout syndrome in nurses in the most varied forms of strategies, with the aim of improving the quality of health of this professional class, and thus, to promote a better quality of work and life for nurses, resulting in a better quality of care for patients.

Review studies in other professional categories have shown that 80% of 25 intervention programs evaluated were useful in reducing burnout in general or in one of its components [18].This result is confirmed in interventions focused on prevention or treatment, or those aiming to improve the strategies of occupational stress management. According to these studies, teaching workers to manage the stressors in their work environment allows a change in the perception on certain stressful characteristics of work, thereby reducing somatic complaints. Another similar intervention identified that the greatest effect of intervention occurs with increased behavioral control over work [18]. In this way, the authors concluded that teaching the participants on the management of stressors in their work environment would allow them a change in the perception of these stressors, thereby reducing the tensions and complaints related to occupational diseases.

## 4 DISCUSSION

The amount of stress and burnout experienced by nurses is suggested to be a function of the nurses’ work environment and coping resources. The way a stressful event is perceived depends on individual characteristics, resilience, and coping skills [19]. The interventions analyzed were effective in reducing burnout levels, based on the success of the interventions in meeting the objectives proposed. The success of the interventions was based on repetition through the method they delineated, from which over time it was possible to reduce levels of stress [5, 20]. The professionals analyzed by the studies reviewed usually worked in a hospital environment. The hospital environment contains stressors, such as death situations, emergencies, and several associated functions that lead the professional to develop a high stress load. This fact increases the chances of occurrence of burnout syndrome [11].

Several strategies can be employed to reduce burnout in nurses. Mealer *et al.* reported a resilience training program consisting of teaching professional techniques for dealing with cognitive behavior and increasing their resilience to the challenges demanded by the nursing profession. They proposed that resilience can be taught, developed, and strengthened through coping skills training [5]. Pipe *et al.*; Oman, Hedberg, and Thoresen, suggested that meditation is also a strategy that helps reduce stress, especially in nurses [14]. The strategy is based on the perception of stress, and with meditation practice, stress becomes easier to deal with, regardless of the source of the stress. Yoga practitioners, according to Alexander *et al.*, after eight weeks of practice, reported less emotional exhaustion and depersonalization, thus demonstrating the effectiveness of this type of action to combat burnout in nurses [6].

Mackenzie, Poulin, and Seidman-Carlson, presented a mental health program for nurses: audiorecorded mental-exercise exercises, which the nurses listened to for 10 minutes a day, five days a week, for four weeks; this intervention demonstrated effectiveness in reducing burnout [12]. A professional identity development program was conducted by Sabancıogullari and Dogan. The program focused on developing professional self-image and positive professional thinking, setting professional goals, evaluating the professional self, developing short-term professional goals, brain programming, and developing successful strategies to increase job satisfaction. Even online interventions showed significant results [17]. In a study by Hersch *et al.*, the application of seven intervention modules *via* the web allowed for improvements in the coping strategies normally experienced by nurses [19]. The program consisted of sending e-mails to the nurses, who reported the main stressors of their work environment. The nurses subsequently received directions on how to deal with stress at work.

Markwell *et al.* pointed to such activities as Reiki, Touch of Healing, Therapeutic Massage, Jin Shin, Jyutsu (Art of Release of Stress), and Guided Images, as promising interventions for stress [10]. Another possibility for intervention in the prevention of work stress is centering actions on the meaning of job satisfaction and quality of life. Fillion *et al.* evaluated the impact of this action on palliative care nurses [16]. The evaluated group participated in a pre-test and three months of follow-up. The tests were applied by facilitators (psychologists) who evaluated how much emotions and humor can diminish nurses’ stress. According to Kim and Park, compassion fatigue is a stress type that can evolve to burnout [21]. They proposed a Compassionate Fatigue Program that aimed to reduce the sense of empathy and compassion of nurses in Korea to avoid the emotional stress experienced by practitioners. Ross *et al.* formulated a program to encourage physical activity for nurses, called Nurses Living Fit, in which nurses completed 12 hours of weekly exercise and received information on healthy lifestyles, nutrition, and appropriate hours of sleep [22].

Kutney-Lee *et al.* reported that changes in nurses’ work environment over time may lead to decreased burnout rates and job dissatisfaction [23]. Gasparino and Guirardello [4] found that a work environment that does not favor the professional practice of nurses can benefit the emergence of burnout. In a study carried out with three hospitals in São Paulo, Brazil, the hospital that favored the nurses’ work environment had the lowest rates of burnout. Darban, Balouchi, and Housein [11] concluded that communication skills training decreases the level of burnout in nurses.

Kubota *et al.* observed changes in self-reported confidence, knowledge, and attitudes regarding common psychological problems after the implementation of a program for psychological training in oncological nurses. The nurses were evaluated between pre-intervention and three months post-intervention. Their secondary outcomes were work-related stress reduction and burnout, which were not significant in several cases. The oncological nurses participated in a 16-hour program conducted as two one-day meetings over two consecutive weeks. Another purpose of the program was to train nurses to better serve patients [24].

Ross *et al.* implemented a program of physical activity incentive for nurses called Nurses Living Fit that involved 12 hours of weekly exercise and information provision on healthy lifestyle, nutrition, and adequate sleep. The program was effective in reducing the prevalence of overweight in the participating nurses after the 12-week program ended [22]. Khamisa *et al.* presented the correlation between burnout, work satisfaction, and the general health of nurses; they concluded that strategies for stress management and job satisfaction can lower the burnout level in these professionals [7].

Another instrument of burnout prevention is the cognitive or psychological aluation applied in nurses. Pereira and Gomes applied the following scales to evaluate the stress in health professionals: Demographic Questionnaire, Maslach Burnout Inventory, Human Services Providers questionnaire, Beck Depression Inventory, and Cognitive Assessment Scale. The data were evaluated by psychologists, and statistical analysis was done to assess the level of burnout in nurses. They aimed to intervene with the most stressful factors in the places of work [25]. The study showed the importance of cognitive assessment to shed light on how professionals react to stress situations in their workplace.

Occupational stress in the health field is directly related to specific situations. Issues of relationship, ambiguity and conflict of functions, double working hours, and pressures undertaken by superiors [12], as well as poor working conditions, lack of material resources, and equipment without proper maintenance, contribute to frequent damage or inappropriate improvisations, causing serious errors that compromise patient care [19].

### 5.1 Limitations of the Reviewed Studies

Scientific work on the burnout remains scarce in terms of prevention and control of burnout in nurses, especially as related to actions that can lessen the event. Most of the studies have focused on the actions for combatting burnout in nurses when the professionals already have the syndrome. Studies on disease prevention actions are also insufficient. In addition, the use of different methods to measure Burnout or well-being in nurses also represents a limitation. More studies are needed to identify the possible limitations of the interventions in a given context.

## CONCLUSION

This study focused on strategies to minimize and control burnout symptoms among nursing professionals. The review identified varied interventions encompassing individual, group, and organizational actions, with a significant prevalence of group actions. The results indicated that the strategies used to cope with burnout were, for the most part, effective, with some demonstrating greater success than others. The various intervention strategies presented in this study can be used to reduce the effects of burnout among nurses. From the 30 reviewed studies, the results did not obtain satisfactory improvement in burnout in only three interventions: 1) Psycho-oncological training program (Kubota *et al*.) [24]; 2) Systematic clinical supervision (Palsson *et al.*) [26]; and 3) Basic nursing care (Melchior *et al*.) [29]. The assessments were justified by the following variables: sample size, work environment, and working hours.

## AUTHORS CONTRIBUTION

All authors have agreed on the final version of the manuscript and meet at least one of the following criteria (recommended by the ICMJE [http://www.icmje.org/ recommendations/):

1. Substantial contributions to conception and design, acquisition of data, or analysis and interpretation of data;
2. Drafting the article or revising it critically for important intellectual content.

## Figures and Tables

**Fig. (1):**
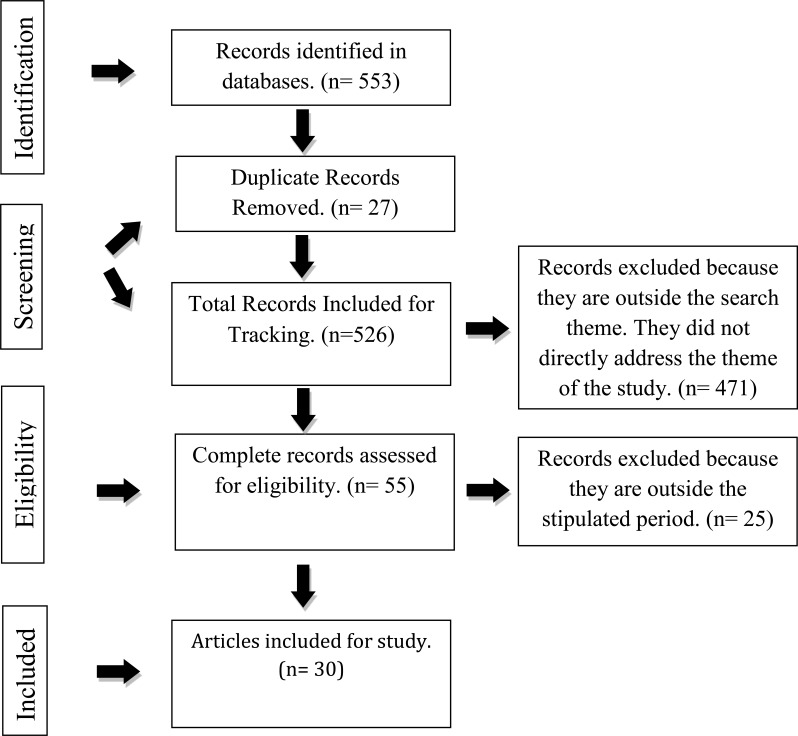
Flowchart showing the selection of studies for the integrative review (Prisma 2009 Flow Diagram).

**Table 1:** Main results found in the articles selected for the review.

**Author and Year**	**Place of Study**	**Nº of Professionals**	**Method Used**	**Prevention / Suggestions of Interventions in Groups**	**Results**
**Afecto, M.C.P. Teixeira, M.B., [27]**	Intensive Unit Care	26	Stress Inventory for Nurses (SIN).	Development of teamwork and improvement courses .	The courses help to minimize the labor stress.
**Alexander, **et al**. [6]**	Institutional Review Board (IRB)	40	Maslach Bournout Inventory	Yoga	The benefits and limited risk of yoga can help employee-level outcomes, including mental health.
**Berg, A.; Hansson, U. W.; Hallberg, I.R., [9] **	Hospital	39	Maslach Burnout Inventory.(MBI)	Nursing supervision and implementation of individually planned care	Boredom and Burnout decreased significantly after the intervention.
**Darban, F.; Balouchi, A.; Housein, S. [11]**	Hospital in Iranshahr(Iran)	60	Jacson and Malach Burnout Inventory	Communication skill training.	The training proved to be effective in reducing Burnout.
**Fillion, **et al**. [16]**	Nurses from Three Regional Districts of Quebec, Canada	109	General Satisfaction subscale	Intervention focused on the meaning of job satisfaction	Nurses have improved their perceptions about their jobs, generating benefits, personal growth and reduced burnout. .
**Gasparino, R.C.; Guirardello, E.B., [4]**	Hospitals (two tertiary and one secondary)	278	Nursing Work Index Revised - Brazilian Version and the Maslach Burnout Inventory .	Changes in the workplace	The management of good professional practices, favor the decrease of Burnout in nurses.
**Günüşen, N. P.; Ustün, B., [18]**	Universitary hospital	108	Maslach’s Burnout Inventory (MBI)	Cognitive coping strategy	Interventions have been shown to decrease Burnout if applied repetitively
**Hersch, **et al**. [19]**	New York Hospital	104	Mental Health Scale	Web-Based Stress Management Program	The results show benefits of the program in reducing stress and Burnout.
**Khamisa, **et al.**, [7]**	Private and public hospitalsin Gauteng province (South Africa)	895	Questionnaires	Strategies to manage stress, improving job satisfaction.	Creating better work environments and adequate work resources will reduce Burnout.
**Kim, Y. A.; Park, J. S., [21]**	Hospitals	32	Validated questionnaire	Program to combat fatigue of compassion	The program was effective in reducing the fatigue of compassion among nurses .
**Kubota, **et al**. [24]**	Aichiprefecture, Japão	96	Maslach Bournout Inventory (MBI)	Psycho-oncological training program	There was no significant result regarding Burnout.
**Kutney-Lee, **et al**. [23]**	137 hospitals in Pensilvania(USA)	67.000	Search by email	Improvements in work environment.	Improvements in the work environment are associated with low stress rates.
**Mackenzie, C. S.; Poulin, P. A.; Seidman-Carlson, R. A., [12]**	Urban hospital	30	Mindfulness-based stress reduction (MBSR)	Mental Attention Training	The training achieved an improvement in Burnout symptoms, relaxation and life satisfaction.
**Markwell, **et al.**, [10]**	Hospital	210	Holistic Care	Reiki, Healing Touch, Therapeutic Massage, Jin Shin,Jyutsu ( Art of release of tensions) and Guided Images.	Holistic interventions in the workplace, relieve stress and provide some relaxation for nurses .
**Mealer, **et al**. [5]**	Intensive Unit Care	27	Malach Bournout Inventory (MBI)	Resilience training program.	Resilience training has improved levels of depression, anxiety, and burnout.
**Melchior, **et al**. [29]**	Primary health care institution	161	Maslach Bournout Inventory (MBI)	Basic Nursing Care	It did not result in significant burnout reduction.
**Moody, **et al**. [30]**	Pediatric oncology clinic	47	MBC, Mindfulness-based Course	Mindfulness-Based Course	The Course based on mental alertness can relieve Burnout symptoms.
**Morita, **et al**. [31]**	Seirei Mikatahara General Hospital	76	MalachBournout Inventory (MBI)	Spiritual Pain Assessment Sheet	The program achieved improved self-confidence of nurses and decreases Burnout.
**Oman, D.; Hedberg, J.; Thoresen, C. E., [32]**	Large hospital in Colorado, USA	58	Maslach Bournout Inventory (MBI)	Meditation	Evidence shows that this program reduces stress and can improve mental health and reduce Burnout,
**Orly, **et al**. [13]**	Regional Hospital of Southern Israel	36	Mental Health Scale	Cognitive-behavioral interventions	The Interventions reduces stress reactions at work.
**O ¨ Zbas¸ A. A.; Tel, H., [33]**	Cancer Hospital	82	Empowerment Scale and Maslach’s Burnout Inventory (MBI)	Psychological Empowerment Program .	The program decreases levels of burnout in nurses .
**Pålsson, M. B., **et al**. [26]**	Districts	33	Karolinska Scale of Personality	Nursing supervision	There was no significant reduction of Burnout
**Pereira, M. M. A.; Gomes, A. R. S., [25]**	Public hospital	153	Stress Questionnaire for Health Professionals (SQHP)	Cognitive evaluation	The Burnout decreased
**Pipe, **et al**. [14]**	Mental health institution	32	Caring Efficacy Scale	Meditation	Participants in the meditation course had significantly more improvements in stress reduction.
**Poulsen, **et al**. [34]**	Hospital oncology industry	70	Recovery Experience Questionnaire (REQ)	Interventional Workshop	There was no significant reduction of Burnout
**Redhead, **et al**. [20]**	Mental health unit.	42	Maslach Bournout Inventory (MBI)	Psychosocial intervention	The program resulted in a small but positive change in the levels of Burnout in nurses .
**Ross, **et al.**, [22]**	Harvard Medical School and Brighamand Women’Hospital	40.000	Monitoring of healthy behavior of nurses	Incentives to exercise, eating a healthy diet, reducing stress and improving interpersonal relationships .	Recommendation for nurses by assuming a healthy lifestyle to reduce stress.
**Sá,A.M.S.;** **Silva, P. O. M.; Funchal, B., [8]**	Public hospital	52	Maslach Bournout Inventory (MBI)	Public management policies in nursing	The results show that satisfaction with the environment reduces emotional exhaustion at work.
**Sabancıogullari, S.; Dogan, S., [15]**	Universitary hospital	310	Professional Self-Concept Inventory (PSCI)	Professional identity development program	The program increased the development of professional identity and decreased Burnout.
**Zadeh, **et al.** [35]**	National Institutes of Health	126	10- Welfare Program Sessions.	Wellness program for nurses. .	It is necessary to provide supportive care, for coping with Burnout on an ongoing basis.

**Table 2:** Distribution of the types of interventions by categories adapted from Salanova & Llorens (2008) [17].

**Type of Intervention**	**Category Type**	**(%)**
-Yoga; - Nursing supervision *(two authors);* - Web-Based Stress Management Program; - Individual meditation; - Cognitive evaluation; - Cognitive coping strategy;	Individual	23,33
- Communication skill training; -Intervention centered on the meaning of work; - Compassionate Fatigue Combat Program; -Program of psycho-oncological training; - Mental attention training *(two authors);* -Intervention Office; - Spiritual Pain AssessmentSheet; - Cognitive-behavioral interventions; - Psychological empowerment program; -Intervention Office; - Psychosocial intervention; -Incentives to exercise, consumption of diet -relaxable, reduction of stress and improvement of interpersonal relationships; - Professional identity development program; -Intervention focused on the meaning of job satisfaction; - Meditation Course.	Grupal	53,33
-Development of teamwork and improvement courses39; - Changes in Work Environments *(two authors);* -Strategies for managing stress, improving job satisfaction5; -Reiki, Healing Touch, Therapeutic Massage25; - Basic nursing care30; - Public management policies in nursing6.	Organizational	23,33
**Total**		**100**

**Table:** Authors contribution table:

**Criteria**	**Author Initials**
Made substantial contributions to conception and design, or acquisition of data, or analysis and interpretation of data;	SO
Involved in drafting the manuscript or revising it critically for important intellectual content;	SO, VN, SL
Given final approval of the version to be published. Each author should have participated sufficiently in the work to take public responsibility for appropriate portions of the content;	SO, VN, SL, MG
Agreed to be accountable for all aspects of the work in ensuring that questions related to the accuracy or integrity of any part of the work are appropriately investigated and resolved.	SO, VN, MG
